# Robotic Single‐Port Versus Robotic Single‐Site Hysterectomy in Early Endometrial Cancer: A Case Control Study

**DOI:** 10.1002/rcs.70107

**Published:** 2025-09-15

**Authors:** Riccardo Vizza, Giacomo Corrado, Valentina Bruno, Ermelinda Baiocco, Pier Carlo Zorzato, Stefano Uccella, Enrico Vizza

**Affiliations:** ^1^ Department of Obstetrics and Gynecology AOUI Verona University of Verona Verona Italy; ^2^ Dipartimento Scienze della Salute della Donna del Bambino e di Sanità Pubblica UOC Ginecologia Oncologica Fondazione Policlinico Universitario A. Gemelli IRCCS Rome Italy; ^3^ Gynecologic Oncology Unit Department of Experimental Clinical Oncology IRCSS‐Regina Elena National Cancer Unit Institute Rome Italy

**Keywords:** Da Vinci SP, robotic hysterectomy, SP surgery

## Abstract

**Objective:**

To compare surgical outcomes of robotic single‐port (RSPH) versus single‐site (RSSH) hysterectomy in early‐stage endometrial cancer.

**Methods:**

This is a retrospective case‐control study, comparing surgical outcomes of RSPH (Cases) and RSSH (Controls) in early‐stage endometrial cancer.

**Results:**

Twenty‐five women who underwent RSPH from June 2024 to November 2024 were matched with 50 historical RSSH controls treated at the same institution by the same surgical team between December 2011 and September 2014. Operation time was similar: 110 min in RSPH and 99 min in RSSH (*p* = 0.76). Blood loss was 50 mL in RSPH and 60 mL in RSSH (*p* = 0.14). Hospital stay was shorter in RSSH (3.5 days in RSPH and 3 days in RSSH, *p* = 0.001).

**Conclusions:**

Our study confirms the safety and feasibility of RSPH for endometrial cancer without major differences from the RSSH in terms of surgical outcomes.

## 1 Introduction

Robotic‐assisted surgery has been shown to provide similar benefits to those of conventional laparoscopic approaches for hysterectomy in endometrial cancer. Within the realm of robotic surgery, two alternatives to conventional robotic hysterectomy have been developed: robotic single‐port hysterectomy (RSPH) and robotic single‐site hysterectomy (RSSH). Both approaches enhance the cosmetic outcomes of minimally invasive procedures while reducing the potential risks linked to multiple incisions [1, 2].

DaVinci SP is a specialised variant of the Da Vinci surgical system tailored for single‐port surgery. It utilises a single robotic arm that passes through one incision. DaVinci Single‐Site is a set of single‐use instruments and accessories developed in 2010 by Intuitive Surgical Inc., specifically designed for the DaVinci multiport platform, allowing all instruments from the different arms to be inserted through a single incision.

Between these two surgical techniques, RSPH is the most recent and there is scant literature available on it. Recently, several research groups, including ours, have demonstrated the feasibility of a single‐port transumbilical approach for low risk endometrial cancer, but the present evidence is limited to small case series [3]. Moreover, RSPH has some limitations and remains a challenging procedure due to its technical difficulties. The aim of this study is to compare robotic single‐port hysterectomy (RSPH) versus robotic single‐site hysterectomy (RSSH) in early‐stage endometrial cancer in terms of perioperative outcomes.

## 2 Materials and Method

This is a retrospective case‐control study, comparing surgical outcomes of robotic single‐port hysterectomies (RSPH, Cases) and robotic single‐site hysterectomies (RSSH, Controls) in early‐stage endometrial cancer patients. The study received ethical approval from the Regina Elena National Cancer Institute of Rome, Italy (RS: 322/IRE/25). Written informed consent for either RSPH or RSSH was obtained from all participants in accordance with local and international ethical standards (declaration of Helsinki) [4]. All cases were performed on DaVinci SP surgical system (Intuitive Surgical Inc. ; 1266 Kifer Road, Building 101, Sunnyvale; CA) and the surgical team was composed of the primary surgeon (E.V.), a bedside assistant, a circulating nurse and a scrub technician specialised in robotic procedures.

### 2.1 Study Design

Patients diagnosed with endometrioid‐type endometrial carcinoma (EGCC) by the International Federation of Gynaecology and Obstetrics stage IA‐IB were eligible for RSPH or RSSH. Other eligibility criteria included: tumour grades well (G1) or moderately differentiated (G2) at pre‐operative biopsy; adequate vaginal access and uterine size < 12 weeks gestation; adequate bone marrow reserve (absolute granulocyte count ≥ 2000/mL, platelet count ≥ 100000/mL); and adequate cardiac, hepatic, and renal function; and ECOG performance status of 2 or less.

Patients were excluded from surgery if they had any of the following: significant cardiopulmonary disease or contraindications for prolonged Trendelenburg position; prior pelvic or abdominal radiotherapy; or severe hip disease limiting dorsolithotomy position. Previous abdominal surgery did not disqualify patients from the robotic approach.

Clinical variables assessed included patient age, body mass index (BMI), FIGO stage, and tumour grade. Additionally, surgical margin status and length of hospital stay were evaluated The operation time was categorised into three phases: (1) docking time, the time from the moment of port placement until the second robotic arm is mounted to the corresponding cannula; (2) console time, the duration during which the surgeon actively controlled the instruments; and (3) operation time, measured from skin incision to closure. Intraoperative data collected comprised complication rates, estimated blood loss, and haemoglobin levels before and 24 h post‐surgery. Blood transfusions were given when haemoglobin dropped to ≤ 7 g/L. Postoperative complications were assessed both short term (within 30 days) and long term (beyond 30 days), focussing on surgical, cardiac, pulmonary, and other events.

### 2.2 Surgical Procedure

All patients received antibiotic prophylaxis with intravenous Augmentin (2.2 g) and perioperative low molecular weight Enoxaparin (40 mg/24 h, subcutaneously). The vaginal cavity was prepared using a povidone‐iodine solution, and a Foley catheter was inserted into the bladder. Intraoperative lower extremity sequential compression devices were applied for venous thrombosis prophylaxis. All procedures were performed under general endotracheal anaesthesia.

#### 2.2.1 RSPH

After indocyanine intracervical injection, we used swabs to buffer the vagina. The cervix is closed with a modified tenaculum called ‘simple nebs arising incision landmark’ (SNAIL) [5] and no uterus manipulator device is used.

**FIGURE 1:**
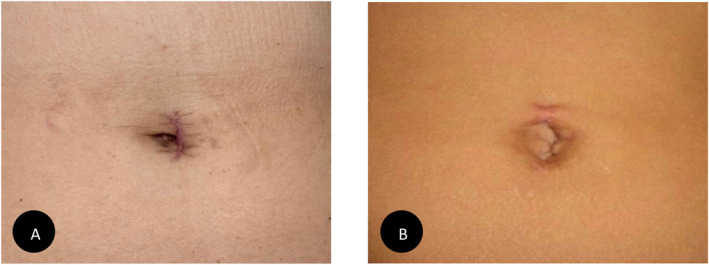
Postoperative surgical scars following single‐port (SP) and single‐site (SS) procedures. (A) SP surgical scar, (B) SS surgical scar.

The procedure begins with initial access through a 2.5 cm incision made at the lower edge of the umbilicus, extending down to the fascia, which is then opened along the body's longitudinal axis (Figure 1).

**FIGURE 2:**
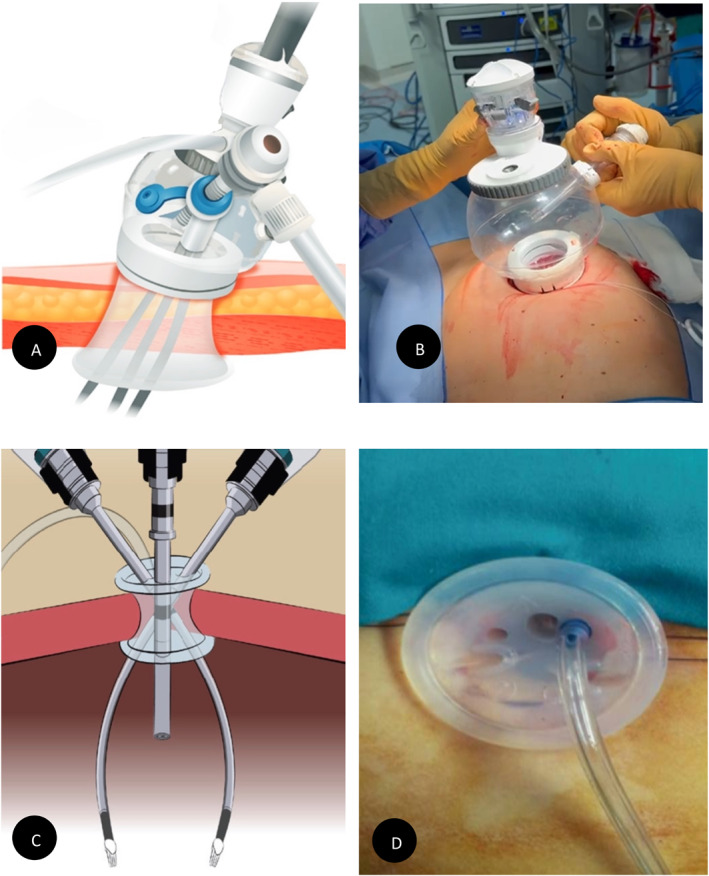
Comparison of single‐port (SP) and single‐site (SS) access techniques. (A) Intuitive access port positioned in the umbilical incision, (B) Single‐site port positioned in the umbilical incision, (C) Schematic drawing of single‐port access, and (D) Schematic drawing of single‐site access.

The leading edge of the folded port is introduced into the incision with a downward motion while countertraction is provided by retractors within the incision. An Intuitive Access Port of small size is inserted into the incision and pneumoperitoneum is created by insufflating the abdominal cavity to a pressure of 12 mm Hg (Figure 2A,B). Patients are then placed in the steep Trendelenburg low‐lithotomy position, and the Da Vinci SP robot is docked laterally to the patients on the left side.

The Intuitive Access Port has four different channel ports: in the superior channel, a three‐dimensional 8.5‐ mm optics is inserted; in channels 1, 2 and 3 are inserted a Cadiere Forceps, a Maryland Bipolar forceps and Monopolar Scissors (MS), respectively. All instruments, including the camera, are articulating.

The entire abdominal cavity is thoroughly examined to detect any suspicious peritoneal lesions that might preclude completion of the procedure using the robotic SP approach: the inspection is performed by inserting only the optics into the port. Afterwards, the remaining instruments are inserted, and the surgical procedure begins. Following the resection of the round ligament and incision of the retroperitoneum along the path of the iliac external vessels, the ureter is identified; a window is opened between the ovarian pedicle above and the ureter below, and ovarian pedicles are coagulated with Maryland Bipolar and resected with MS. Then, we develop the retroperitoneal spaces (paravesical space, lateral and medial pararectal space) bilaterally, identify the sentinel node on each side, and remove it. Afterwards, a type A hysterectomy is performed as described by Querleu and Morrow and uterine vessels are coagulated with Maryland Bipolar and resected with MS. Colpotomy is performed using MS. The uterus and adnexal structures were removed transvaginally and submitted for definitive histopathological analysis. The vaginal cuff is closed with a continuous suture using V‐Loc and a needle driver in place of the MS. In case of no mapping on one or both pelvic sides, systematic pelvic lymphadenectomy was performed.

The arrangement of the instruments is the same as for hysterectomy. Lymphadenectomy is divided into three steps:

- First step: the surgeon prepares the medial limit of the lymphadenectomy starting from the common iliac artery following the hypogastric artery and the obliterated umbilical artery.
- Second step: the lateral limit is prepared by detaching pelvic vessels from the muscular surface of the pelvis
- Third step: the periadventitial plate of the common iliac artery and the external iliac artery is incised in a centrifugal direction and the lymph nodes are removed ‘en bloc’ up to the obturator nerve. The lymph nodes are inserted in an endobag and removed through the vaginal canal before the vagina is sutured.

#### 2.2.2 RSSH

All the procedures were performed using the da Vinci Si Surgical System (Intuitive Surgical Inc. , Sunnyvale, CA). A single 2.5 cm umbilical incision accommodated a multi‐channel system along with two single‐site robotic 5 mm instruments (Figure 2C,D). Additionally, a 3‐dimensional, high‐definition 8.5 mm endoscope and a 5 mm accessory instrument were utilised. Class A hysterectomy including bilateral salpingo‐oophorectomy was performed following the Querleu and Morrow [6]. A detailed description of the robotic single‐site approach used in all procedures has been previously reported [7].

#### 2.2.3 Statistical Analysis

Demographic and clinical characteristics of the patients were summarised using descriptive statistical methods. The Mann–Whitney and Fisher exact tests were used to compare continuous and categorical variables, respectively. A *p*‐value < 0.05 was considered statistically significant. All statistical analyses were conducted using SPSS software SPSS software (SPSS version 21.0, SPSS Inc., Chicago, Illinois, USA).

## 3 Results

A total of 25 women who underwent RSPH from June 2024 to November 2024 were matched with 50 historical RSSH controls treated at the same institution by the same surgical team between December 2011 and September 2014.

Table 1 reports the clinical, demographic and pathological characteristics of the two groups. The two groups had similar median age and BMI. There were no statistically significant differences in comorbidity rates between the two groups. Final pathological findings were comparable in terms of tumour histology. Significant statistical differences were observed in terms of FIGO stage and tumour grading. Notably, the RSSH group had a substantially higher percentage of stage IA cases compared with the RSPH group. Moreover, the RSPH group had a higher grading compared to the RSSH group.

**TABLE 1:** Clinical characteristics.

Characteristics	RSPH (patients = 25)	RSSH (patients = 50)	*p* value
Age Median (range)	61 (33–83)	62 (35–84)	0.593
BMI (kg/m^2^) Median (range)	28.44 (17.3–45.8)	26.64 (17.8–51.5)	0.399
Histology			
Adenocarcinoma	25 (100)	49 (98)	0.999
Adenosquamous	0 (0)	1 (2)	
FIGO stage			
IA	13 (52)	40 (80)	0.016
IB	12 (48)	10 (20)	
Grading			
G1	4 (16)	24 (48)	0.031
G2	18 (72)	21 (42)	
G3	3 (12)	5 (10)	

Abbreviations: BMI, body mass index; RSPH, robotic single port hysterectomy; RSSH, robotic single site hysterectomy.

Operative details and outcomes are summarised in Table 2. Patients in the RSPH group had a similar median operative time compared to the control group (110 vs. 99 min, respectively; *p* = 0.761). The median blood loss was 50 mL in the RSPH group and 60 mL in the RSSH group. This difference was not statistically significant (*p* = 0.141). We registered no intra‐operative complications in the two groups. No cases required conversion to either laparotomy or conventional laparoscopy. The median length of hospital stay was 3.5 days in the RSPH group and 3 days in the RSSH group (*p* ≤ 0.05).

**TABLE 2:** Surgical outcomes.

Characteristics	RSPH (patients = 25)	RSSH (patients = 50)	*p* value
Operative time (min.) Median (range)	110 (55–215)	99 (45–200)	0.761
Blood loss (mL.) Median (range)	50 (10–230)	60 (10–250)	0.141
Hb drop (gr/dl) Median (range)	1.0 (0.0–3.46)	1.5 (0.1–3.5)	0.104
Major intraoperative complications (%)	0	0	—
Major early postoperative complications (%)	4	4	0.999
Major late postoperative complication (%)	0	0	—
Blood transfusion (%)	0	4 (2)	0.691
Conversion to laparoscopy (%)	0	0	—
Conversion to laparotomy (%)	0	0	—
Re‐intervention	1	1	0.999
Hospital stay (day) Median (range)	3.5 (2–11)	3 (2–6)	0.003

Abbreviations: RSPH, robotic single port hysterectomy; RSSH, robotic single site hysterectomy.

The difference in the incidence of early and late complications among groups was not statistically significant. On the first postoperative day, a patient with RSPH with a history of thrombocytopaenia presented with a hemoperitoneum of 450 mL and hypotension. Laparoscopy was accomplished and bleeding from a right vaginal artery was identified and fixed with bipolar cautery. The patient was discharged on the 11th day after the robotic surgery in good general condition. We observed one grade 2 and one grade 3 postoperative complications according to CTCAE v4.03 [8] in the RSSH group. One patient had fever that required prolonged antibiotic therapy. The second had hemoperitoneum that required a laparoscopic re‐intervention. Median Hb drop was 1.0 gr/dl in the RSPH group and 1.5 gr/dl in the RSSH group, with no significant difference. Two blood transfusions in the RSSH group were registered but the difference did not reach statistical significance. These two transfusions were performed due to moderate anaemia before surgery and not because of excessive blood loss during surgery (Table 2).

## 4 Discussion

Our study shows that RSPH with sentinel node is feasible in the setting of endometrial cancer patients and preliminary outcomes appear superimposable to a historical cohort of patients who were treated with robotic single‐site surgery for the same malignancy.

Single‐port procedures represent an important advancement in robotic surgery. The technique allows to minimise surgical trauma to the abdominal wall while maintaining comfortable operability in the abdominal cavity. The first approach to this technique was laparo‐endoscopic single‐site surgery (LESS), initially reported by Hirano et al. [9] in 2005 with a retroperitoneoscopic adrenalectomy performed through a single incision using standard laparoscopic instruments. In 2009, Langebrekke et al. [10] performed the first single‐port laparoscopic hysterectomy. However, LESS presents significant technical challenges, including instrument crowding and limited manoeuvrability.

To address these limitations, the robotic single‐site (SS) approach was introduced as an alternative. This method combines the aesthetic benefits of a single incision with enhanced dexterity, thanks to wristed instruments, tremor filtration, improved ergonomics (including the avoidance of using with the right hand the instrument on the left and vice versa, as it happens for LESS), and a three‐dimensional visualisation system [11]. In 2009, Fader et al. [12] performed the first robotic single‐site hysterectomy. Since then, studies have demonstrated the feasibility of robotic single‐site surgery (RSSH) in treating endometrial cancer [7, 13, 14]. However, despite its advantages, RSSH still has several drawbacks: it uses straight, wristed‐mounted instruments with limited instrument articulation, external collisions, and reduced versatility.

The da Vinci Single Port system by Intuitive Surgical Inc. (Sunnyvale, CA, USA) was approved for gynaecologic surgery in South Korea in 2019 and in Japan in 2022. In the United States, while the SP system received FDA approval in 2019 for select otolaryngology and urology procedures, approval for gynaecologic applications remains pending. In the European Union, SP was approved for gynaecological procedures in 2024. Since 2019, SP surgery has been successfully integrated into clinical practice for various procedures, including cholecystectomy, colorectal surgery, and urological surgery, with encouraging preliminary results [1, 15].

Currently, there is limited literature on the use of the da Vinci SP system gynaecologic oncology. Most studies originate from South Korea and Japan, where early adoption of the technology has allowed for initial clinical assessments [16, 17, 18]. More recently, two European studies have also been published: one demonstrated that the implementation of the da Vinci SP platform in endometrial cancer did not increase conversion rates or negatively affect perioperative outcomes [19], while another reported that single‐port robotic surgery appears to be a promising and well‐tolerated approach for surgical staging of early‐stage ovarian cancer in selected patients [20].

Robotic single‐port (SP) surgery represents a decisive evolution of the SS approach, overcoming many of its limitations, such as loss of triangulation, instrument crowding, poor visualisation, and ergonomic challenges. The SP system features a multi‐articulated camera and instruments (with two points of articulation within the body), allowing for improved angulation towards the surgical field. In general, the SP system offers greater freedom of movements, potentially leading to enhanced surgical precision and reduced surgeon fatigue. While both SP and SS systems offer improved cosmesis through a single incision, once well established in clinical practice, the SP system may provide superior results due to enhanced instrument control and reduced external interference.

Thanks to our direct experience with both techniques, we observed these differences firsthand. However, despite these technical improvements, our findings indicate that surgical outcomes between the two platforms remain comparable. This similarity may be due to the learning curve associated with the SP system, as the primary surgeon had no prior experience with this platform. We anticipate that as operator proficiency increases, future independent studies with more accumulating experience will likely demonstrate superior outcomes with SP surgery. Additionally, larger patient cohorts may reveal statistically significant differences in blood loss and hospital stay, further highlighting the advantages of the SP system.

The strength of our study is that it is the first to directly compare the robotic single‐port and single‐site approaches in gynaecologic surgery. Furthermore, having a single primary surgeon perform all procedures ensures consistency and eliminates bias related to operator experience. From 2011 to the present, perioperative pathways have gradually evolved; however, these changes are unlikely to have significantly affected the comparability of our patient groups. Importantly, the surgical procedure itself has remained consistent, as all patients underwent a Type A radical hysterectomy according to Querleu and Morrow [6], which reduces the risk of bias related to temporal variations in surgical technique. The main difference between 2011 and current practice concerns the use of indocyanine green (ICG) for sentinel lymph‐node mapping: in 2011 this approach was still considered experimental and not yet standardized. The small sample size is a limitation, and we are prospectively collecting data, with the aim to conduct future studies with larger populations to strengthen our findings.

In conclusion, Robotic SP surgery is a safe and feasible option for treating endometrial cancer. To fully understand the benefits of this new system, larger comparative studies evaluating robotic SP against other surgical approaches are necessary. As experience with SP technology grows, it has the potential to further improve patient outcomes in minimally invasive gynaecologic surgery.

## Ethics Statement

*IRB Approval*: This study was approved by the Ethics Committee ‘Lazio Area 5’ on April 8, 2025. Approval number: 322/IRE/25.

## Conflicts of Interest

Enrico Vizza serves as a proctor for Da Vinci surgical system training courses organized by Intuitive Surgical Inc. However, Intuitive Surgical Inc. had no role in the study design, data collection, analysis, interpretation, manuscript preparation, or decision to publish this work. The other authors declare that they have no conflict of interest.

## Permission to Reproduce Material From Other Sources

No material from other sources requiring permission has been used in this manuscript.

## Data Availability

Data are available from the authors upon reasonable request.
